# Applicability of computed tomography and rhinoscopy in the diagnosis and monitoring of the treatment of epistaxis in a dog

**DOI:** 10.29374/2527-2179.bjvm008624

**Published:** 2025-01-02

**Authors:** Matheus Daudt Matos, Flávia Silva Raja Gabaglia Toledo, Luana Menezes Rocha, Alexandre José Rodrigues Bendas

**Affiliations:** 1 Veterinarian, MSc. Universidade Estácio de Sá. Campus Vargem pequena, Rio de Janeiro, RJ, Brazil; 2 Veterinarian, MSc. Intergávea Rio de Janeiro, Rio de Janeiro, RJ, Brazil; 3 Veterinarian, TeleLaudos Veterinários, Belo Horizonte, MG, Brazil; 4 Veterinarian, DSc. Departamento de medicina e cirurgia veterinária, Instituto de Veterinária, Universidade Federal Rural do Rio de Janeiro. Seropédica, RJ, Brazil.

**Keywords:** neoplasia, image, endoscopy, epistaxis, neoplasia, imagem, endoscopia, epistaxe

## Abstract

Epistaxis is defined as bleeding from the nasal cavity and can be related to systemic causes leading to coagulation disorders, most commonly hemoparasitosis, or to localized changes in the nasal cavity itself (e.g., intranasal neoplasms). Transmissible venereal tumors (TVT) are malignant round cell neoplasms characterized by an anomalous proliferation of tumor cells disseminated mainly by direct contact between animals. Although transmitted sexually, transmission through contact with mucous membranes and skin tissue can also occur, including the nasal cavity. Although rare, it can have significant clinical implications because it is difficult to diagnose. The diagnosis is made by physical examination, imaging tests (computed tomography and rhinoscopy), and histopathological analysis, which is the gold standard. Treatment is based on the use of chemotherapeutic agents, with vincristine as the drug of choice. This study reported a case of intranasal TVT in a 4-year-old French bulldog and addressed its clinical characteristics, diagnosis, and treatment. It also reported the importance of early recognition of the condition and changes in imaging tests to better understand and manage this atypical presentation.

## Resumo

A epistaxe é definida como hemorragia da cavidade nasal, podendo estar relacionada a causas sistêmicas que levem à distúrbios de coagulação, sendo mais comum as hemoparasitoses ou alterações localizadas na própria cavidade nasal como as neoplasias intranasais. O Tumor Venéreo Transmissível (TVT) é uma neoplasia de células redondas, maligna, caracterizada por uma proliferação anômala de células tumorais disseminadas principalmente por contato direto entre animais. Embora transmitido de forma sexual, a transmissão pelo contato com mucosas e tecido cutâneo também pode ocorrer, incluindo a cavidade nasal. O TVT intranasal, apesar de raro, pode apresentar implicações clínicas significativas devido à dificuldade de diagnóstico. O diagnóstico é realizado por meio do exame físico, exames de imagem (tomografia computadorizada, rinoscopia) e tem como padrão ouro a análise histopatológica. O tratamento é baseado na utilização de agentes quimioterápicos, sendo a vincristina a droga de eleição. O presente trabalho tem como objetivo relatar um caso de TVT intranasal, em um cão de quatro anos de idade, da raça buldogue francês, abordando as características clínicas, diagnóstico e terapêutica. Relata-se ainda, a importância do reconhecimento precoce da condição e as alterações nos exames de imagem para melhor compreensão e manejo dessa apresentação atípica.

## Introduction

The presence of bloody nasal secretions can be related to systemic disorders or factors in the nasal cavity itself (Barbosa & Campos, 2024). Systemic diseases, such as coagulation disorders, vasculitis, systemic arterial hypertension, polycythemia, and blood hyperviscosity, are frequently involved. Nasal diseases associated with epistaxis include trauma, foreign bodies, neoplasms, and chronic rhinitis (Cohn, 2020).

The most common causes of epistaxis in middle-aged to older dogs are intranasal tumors, and the most common neoplasias are adenocarcinomas and sarcomas (Fernandes et al., 2024). Clinical signs associated with intranasal tumors include nasal discharge, facial deformity, and dyspnea (Costa et al., 2023; Ojeda et al., 2018; Singh & Sood, 2016).

Transmissible venereal tumors (TVT) are the oldest tumors in the world and are among the main neoplasms that affect dogs (Daleck & Denardi, 2016; Dagli, 2019). Currently, TVT are classified as round cell neoplasms (Hedlund, 2005) in the same group as mastocytomas, basal cell carcinomas, histiocytomas, and lymphomas (Vermooten, 1987). It is a transmissible cellular neoplasm that affects dogs, mainly those that live a stray life and are of reproductive age (Tinucci-Costa & Castro, 2016). Although most cases occur in the external genital region, some reports show the possibility of experimental transplantation (Trompieri-Silveira et al., 2009).

TVT transmission occurs through the inoculation of viable tumor cells during physical contact, which includes not only mating, but also contact with nasal secretions or other mucous membranes (Oliveira et al., 2023), mainly through the licking and sniffing habits of dogs (Oliveira, 2019; Park et al., 2006; Strakova & Murchison, 2014), which can lead to the implantation of tumor cells in skin lesions, the eyes, nasal cavity, and oral cavity (Silva et al., 2022; Tinucci-Costa & Castro, 2016). Genital or extragenital lesions present as single or multiple masses, usually friable, which, depending on their location, can lead to deformation of the affected organ, mainly the nasal cavity (Brandão et al., 2002; Oliveira, 2019).

In intranasal TVT, neoplasia presents primarily with respiratory symptoms (e.g., nasal secretion, epistaxis, and respiratory difficulty), which can be confused with other nasal diseases (Costa et al., 2021). The diagnosis of intranasal TVT is made based on clinical signs, complemented by cytological/histopathological examinations such as fine-needle aspiration and biopsies, which identify the tumor cell characteristics of this neoplasia (Lima et al., 2020). Histopathological examination revealed the presence of large cells with clear cytoplasm and rounded nuclei; Giemsa staining is often used for identification (Pereira et al., 2021).

Chemotherapy with vincristine sulfate has been shown to be effective in controlling cell proliferation and is the treatment of choice for TVT, including intranasal cases (Souza et al., 2022). Typically, weekly applications are used at a dose of 0.025 mg/kg to 1 mg/kg or 0.5 mg/m^2^ IV, weekly, for 3−6 weeks, with a 7-day interval between doses (Fossum, 2005; Ramadinha et al., 2016; Tinucci-Costa & Castro, 2016). The prognosis is generally considered favorable, with complete tumor remission (Ganguly et al., 2013; Tinucci-Costa & Castro, 2016). However, in some cases of resistance to chemotherapy, radiotherapy has also been applied (Rocha et al., 2023). The combination of therapeutic approaches contributes to better results, although the prognosis depends on the stage of the disease and individual response to treatment.

Despite these advances, intranasal TVT requires further study to fully understand its pathogenesis and develop better therapeutic strategies. The current literature suggests that early management and adequate diagnosis are crucial for therapeutic success and control of tumor spread (Carvalho et al., 2022). This study aimed to report the importance of the association between computed tomography (CT) and rhinoscopic examinations in the diagnosis and therapeutic monitoring of canine intranasal TVT.

## Case report

A 4-year-old French bulldog presented to a private veterinary clinic in the city of Rio de Janeiro with the main complaint of epistaxis in the right nostril. He had an ideal weight for the breed, with normal mucous membranes, no evidence of ectoparasites, and other parameters within normal limits. At the time of consultation, blood was collected for complete blood count and serum biochemistry tests. A complete blood count revealed thrombocytopenia, and tests for antibodies against *Anaplasma spp* (*A. phagocytophilum, A. platys*), *Ehrlichia spp* (*E. canis* and *E. ewingii*)*,* *Borrelia burgdorferi* and *Dirofilaria immitis* antigens (SNAP test 4Dx^®^ Idexx) were performed. The samples were tested positive for the *Anaplasma* and *Ehrlichia* antibodies.

Based on this result, doxycycline (5 mg/kg, BID for 28 days) and prednisone (0.5 mg/kg, SID for 5 days) were prescribed, but the animal returned for treatment after 2 weeks with epistaxis in the left nostril and a new blood count was performed, which revealed leukocytosis (23,900 cells/mcl; reference value: 6,0000−17,000) and platelets within the normal range (457,000 cells/mcl; reference value: 150,000−500,000). CT of the skull and rhinoscopy were then performed to determine the cause of the bleeding.

Cranial tomography, with emphasis on the nasal cavity, was performed with the acquisition of cross-sectional images on a 16-channel multislice device (Ge revolution^®^), with sequences before and after intravenous administration of iodinated contrast based on iohexol 300 mg I/mL.

**Figure 1:**
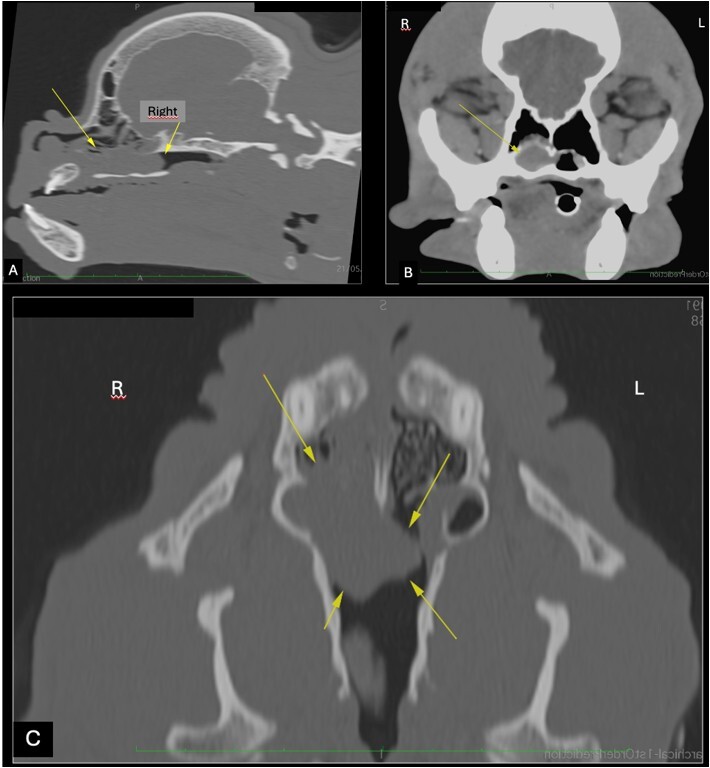
Tomographic images of a French bulldog dog with an intranasal transmissible venereal tumor. **(A)** Sagittal image of the bone philtrum, **(B)** transverse image of the soft tissue philtrum, and **(C)** dorsal image of the bone philtrum. Presence of increased volume of soft tissue inside the right nasal cavity in the ventral aspect in the caudal half (yellow arrows); the nasal turbinates are not delimited.

Tomographic examination revealed an increase in the volume of soft tissues inside the right nasal cavity in the ventral aspect of the caudal half, right nasopharyngeal meatus, choanae, beginning of the nasopharynx, and right sphenoid sinus, suggesting a small part of the right maxillary recess. Measuring approximately 2.8 cm in length × 1.3 cm in height × 1.7 cm in width, where, at the site of this increase in volume, the nasal turbinates were not delimited, possibly due to bone lysis secondary to the injury and discrete fluid content inside the right nasal cavity, rostral to the increase in volume mentioned, with the main differential diagnosis being a neoplastic process (Figure 1). Immediately after the tomographic examination, a rhinoscopy was performed to collect the material for histopathological examination.

**Figure 2:**
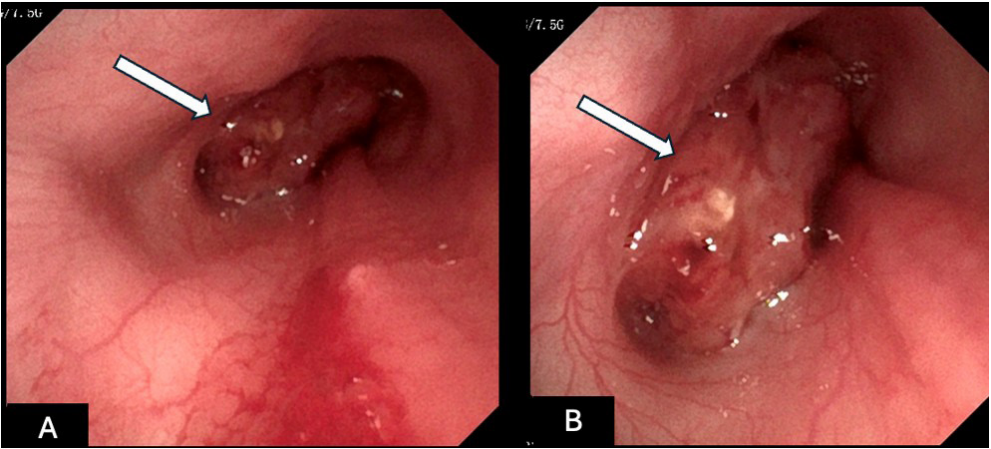
**A and B**-Rhinoscopy of a French bulldog with an intranasal transmissible venereal tumor shows the presence of a neoformation in the right and left choanae region, with an irregular and friable appearance, soft consistency, and reddish-white color (white arrows).

During rhinoscopic examination, a neoformation was observed in the region of the right and left choanae, with an irregular and friable appearance, soft consistency, and reddish-white coloration; the region mucosa was redder than normal (Figure 2). Moderate amounts of mucous secretion were also observed in the bilateral nasal cavity, and aberrant nasal turbinates with irregular appearance and redder mucosa were observed bilaterally. Tissues with irregular appearance and reddish-white coloration were observed in the right ventral meatus. Several biopsy fragments of the neoformation in the nasopharynx were collected. Samples were stored in bottles containing 10% formalin and sent to the histopathology laboratory.

Histopathological examination of fragments of the mass in the nasopharynx and nasal cavity using routine hematoxylin and eosin (H&E) staining revealed tissue presenting with round cell neoplasia diffusely invading the submucosa, characterized by proliferation of round cells with moderate nuclear pleomorphism, evident and central nucleoli, foamy cytoplasm with indistinct edges, and the presence of numerous mitoses, with the sample morphologically suggestive of a transmissible venereal tumor.

**Figure 3:**
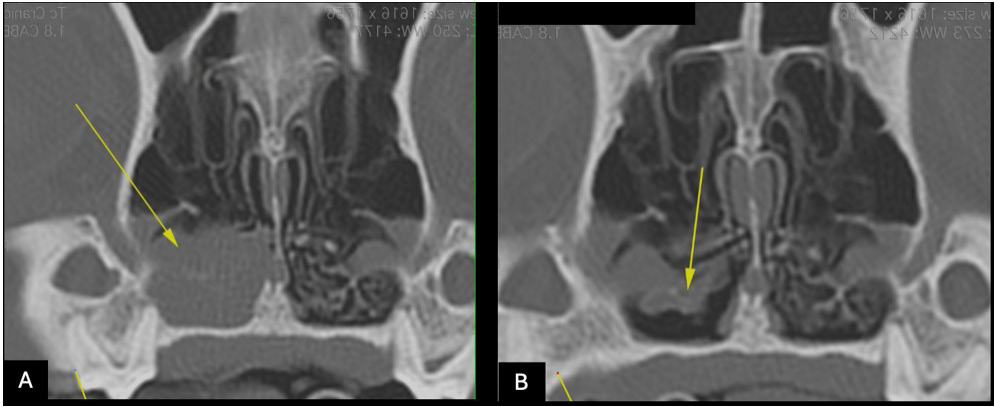
Transverse tomographic images of the bone filter of a French bulldog with an intranasal transmissible venereal tumor before **(A)** and after 6 weeks of treatment with a weekly application of vincristine sulfate **(B).** Presence of amorphous soft tissue content with poorly defined limits, with markedly smaller dimensions after treatment **(B)** (yellow arrows).

Based on the results of the histopathological examination, chemotherapy treatment with vincristine sulfate (0.7 mg/m^2^ weekly for 6 weeks) was instituted and, after 43 days, the patient returned without clinical signs or epistaxis for follow-up of the lesion by tomography examination, where two foci of discrete soft tissue content were observed, amorphous and with poorly delimited limits, located in the right nasal cavity, one of them in the median region of the middle third and the other in the ventral region of the caudal third, suggesting that they are part of the content observed in the previous examination. However, in markedly smaller dimensions, in a subjective evaluation, approximately 10% of the previously delimited content (Figure 3).

Near the ventral region of the nasal cavity, a focal area of ​​absence of nasal conchae was observed, suggesting destruction or even atrophy. Discrete soft tissue content in the ventral aspect of the right nasopharyngeal meatus, with suggestive contact with the medial nasal gland, measuring approximately 0.6 cm in diameter, suggesting that it is part of the content observed in the previous examination. However, in markedly smaller dimensions, it may be less likely to be related to a polyp, granuloma, or aberrant nasal shell with an atypical shape.

**Figure 4:**
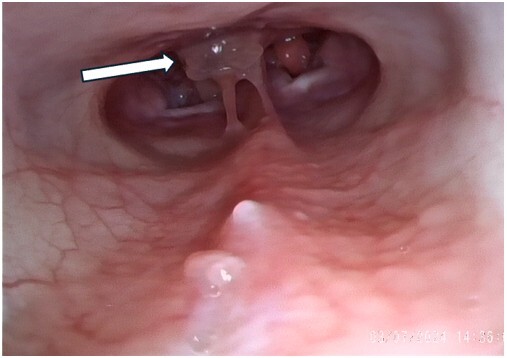
Rhinoscopy of a French bulldog with an intranasal transmissible venereal tumor after 6 weeks of treatment with a weekly application of vincristine sulfate. Rhinoscopic examination revealed a neoformation in the ventral region of the choanae, with an irregular and friable appearance, soft consistency, and reddish-white coloration (white arrow). The neoformation is considerably smaller than the examination before treatment.

In the left nasopharyngeal meatus, aberrant nasal conchae and a tiny structure with soft tissue density measuring approximately 0.36 cm in diameter were observed, suggesting a polyp, granuloma, or atypical shape of an aberrant nasal conchae. Rhinoscopic examination revealed a neoformation in the ventral region of the choanae, close to the nasal septum, with an irregular and friable appearance, soft consistency, and reddish-white coloration. Aberrant caudal nasal turbinates are also observed. The mucosa in the region appeared to be redder than the normal mucosa (Figure 4). The neoformation appeared to be considerably smaller than that observed in the previous examination. A biopsy specimen of the nasopharyngeal neoplasm was collected. The samples were stored in vials containing 10% formalin and sent to the histopathology laboratory.

Histopathological evaluation revealed similar patterns among the samples, highlighting irregular fragments, areas of epithelial hyperplasia, deposition of hemorrhagic foci, and fibrinoid areas. Suppuration points were sometimes observed on the epithelial surface. Intense interstitial lymphoplasmacytic inflammatory migration was observed in the submucosa, with alternating serous points, hemorrhagic foci, and rare suppuration points. The annexed glands appeared hyperplastic with discrete deposition of serous material. Extensive foci of fibrosis and traces of hemorrhage were observed. No evidence of malignancy or infectious agents was observed in any of the samples analyzed, with a diagnostic impression of lymphoplasmacytic hyperplastic rhinitis with areas of fibrosis.

## Discussion

Epistaxis is more common in large male dogs (>26 kg) and aged ≥6 years, which differs from what was observed in the present study in relation to age (4 years) and size (8 kg) (Bissett et al., 2007). Initially, because of nasal bleeding and the age of the animal, the main diagnostic suspicion was the presence of hemoparasitosis as the cause of epistaxis since it is one of the most frequent causes of this type of alteration in young animals and may be related to between 30 and 50% of epistaxis cases (Moreira et al., 2003).

Based on the clinical signs presented by the patient with thrombocytopenia revealed by the blood count, treatment for ehrlichiosis was initiated using doxycycline, the antibiotic of choice for the treatment of rickettsiosis, and prednisone (with the aim of reducing immune-mediated reactions) (Mylonakis et al., 2019). As the patient presented with a new episode of bleeding after 2 weeks of treatment and the platelets were within the normal range for the species, a CT scan of the skull was performed to assess the presence of other causes of bleeding, such as neoplasia and trauma (Bissett et al., 2007). Although the most common intranasal neoplasms (adenocarcinomas and sarcomas) are more common in middle-aged dogs, they were included as possible differential diagnoses (Fernandes et al., 2024). Although the clinical signs associated with intranasal tumors include nasal discharge, facial deformity, and dyspnea, the animal in question only presented epistaxis (Costa et al., 2023; Ojeda et al., 2018; Singh & Sood, 2016).

In cases of cranial evaluation, radiography is limited by the overlap of skull bones and CT is superior to MRI for the evaluation of small bony details, including the presence of bone lysis and periosteal reactions (Giorio et al., 2024). The administration of intravenous contrast (IVC) during CT examination helps accurately localize, describe the margin, and characterize soft tissue masses, providing greater attenuation in (neo) neovascularized structures compared to non-contrast CT images (Crijns et al., 2016).

CT revealed a mass measuring approximately 2.8 cm long × 1.3 cm high × 1.7 cm wide, with bone lysis of the nasal turbinates, suggesting a possible diagnosis of a neoplastic process. However, several other mass-like lesions have similar appearances on CT, including carcinomas, sarcomas, and benign neoplasms (Cissell et al., 2012).

Due to the inability to diagnose the type of tumor using CT, rhinoscopy was chosen because it allows direct visualization of the nasal cavity and collection of material for histopathological examination (Toledo, 2024). The samples were collected by rhinoscopy to determine the type of tumor.

Histopathological examination is considered the standard examination for the differential diagnosis of neoplasms (Fernandes et al., 2024) and allows for the differentiation of the histological type of the sample and the diagnosis of a transmissible venereal tumor. A study carried out in Brazil, with 252 dogs with TVT, found that nasal TVT is the most common extragenital location, representing 9.9% of cases (Costa et al., 2023).

The chosen treatment was chemotherapy with vincristine sulfate, which is the treatment of choice for canine TVT (Costa et al., 2023). CT and rhinoscopy examinations performed 6 weeks after the start of weekly chemotherapy showed favorable evolution of the lesions, and it was possible to determine the patient’s cure through the samples collected by rhinoscopic biopsy because no neoplastic cells were observed.

## Conclusion

This study reports a rare case of epistaxis in the right nostril and shows the importance of a complete diagnostic workout. Detection of tick-borne infection antibodies was misleading, but combining CT, rhinoscopy, and histopathological examinations in diagnosis and follow-up allowed for a proper diagnosis and effective treatment.
